# Influence of endodontic access cavity design on mechanical properties of a first mandibular premolar tooth: a finite element analysis study

**DOI:** 10.1007/s00784-024-05808-x

**Published:** 2024-07-19

**Authors:** Taha Özyürek, Gülşah Uslu, Burçin Arıcan, Mustafa Gündoğar, Mohammad Hossein Nekoofar, Paul Michael Howell Dummer

**Affiliations:** 1 Department of Endodontics, School of Dental Medicine, Bahçeşehir University, Istanbul, Turkey; 2 Department of Endodontics, Faculty of Dentistry, Çanakkale Onsekiz Mart University, Istanbul, Turkey; 3 Department of Endodontics, Faculty of Dentistry, Medipol University, Istanbul, Turkey; 4 Department of Endodontics, School of Dentistry, Tehran University of Medical Sciences, Tehran, Iran; 5 Department of Tissue Engineering, School of Advanced Technologies in Medicine, Tehran University of Medical Sciences, Tehran, Iran; 6 School of Dentistry, College of Biomedical and Life Sciences, Cardiff University, Cardiff, UK

**Keywords:** Endodontic access cavity, Finite element analysis, Mandibular premolar, Minimal invasive endodontic

## Abstract

**Objectives:**

This study aimed to investigate the influence of access cavity designs on the mechanical properties of a single-rooted mandibular first premolar tooth under various static loads using a finite element analysis.

**Materials and methods:**

3-dimensional FEA designs were modeled according to the access cavity designs: an intact tooth (control), traditional access cavity (TEC-I), traditional access cavity with Class-II mesio-occlusal cavity design (TEC-II), conservative access cavity (CEC), ninja access cavity (NEC), caries-driven access cavity (Cd-EC), buccal access cavity (BEC) and bucco-occlusal access cavity (BOEC). After the simulated access cavity preparations, root canal treatment was simulated and three different static loads which mimicked oblique and vertical mastication forces were applied to the models. The stress distribution and maximum Von Misses stress values were recorded. The maximum stress values were obtained on both enamel and dentin under multi-point vertical loads.

**Results:**

The maximum stress values were obtained on both enamel and dentin under multi-point vertical loads. Under all load types, the minimum stress distribution was observed in the control group, followed by CEC, NEC and BEC designs. The highest stress concentration was detected in Cd-EC and TEC-II designs. Under single-point vertical loading, the stress was mostly concentrated in the lingual PCD area, while under multi-point vertical loading, the entire root surface was stress-loaded except for the lingual apical third of the root.

**Conclusion:**

Preserving tooth tissue by simulating CEC, NEC and BEC access cavities increased the load capacity of a single-rooted mandibular first premolar following simulated endodontic treatment.

## Clinical significance

• The BEC design can be considered as a viable alternative in cases involving cervical lesions.

• Oblique and multipoint forces were identified as critical loads impacting the failure probabilities of a root-filled single-rooted first premolar tooth.

## Introduction

Minimally invasive endodontics is a concept based on preserving as much enamel and dentin as possible in the hope of reducing tooth fracture and thus prolonging the survival of the tooth [1, 2]. The concept aims to preserve and retain occlusal tooth structure and pericervical dentin. The pericervical dentin is defined as an area 4 mm above and below the crestal bone [1, 3]. This area is believed to play a critical role in reducing cusp deflection [1]. Technological development allows the preparation of minimal endodontic access cavities where the pericervical region is protected, which has the potential to make this concept more applicable in clinic practice.

Mandibular premolars have been reported to be the most difficult teeth to root fill due to the variations in root canal anatomy of mandibular premolars such as deep splits [4], C-shaped root canals [4], cervical lesions [5], fine ribbon-shaped root canals [6], and multiple canals [7]. To prepare and fill these canal irregularities, adapting the endodontic access cavities to each specific anatomical feature is necessary. In mandibular premolars with a single root canal configuration, the root canal space is accessible using minimally invasive endodontic cavity designs, such as conservative and ninja endodontic cavities, which extend lingually from the central occlusal groove [8]. However, in the presence of challenging root canal anatomy, caries and/or cervical lesions, modified cavity designs may be necessary. Cervical lesions, which can be defined as both carious and non-carious defects, are the most prevalent on the buccal surface of mandibular premolars [5]. In these cases, access to the root canal space can be created with buccal access cavity designs (BEC). It has been reported that if these lesions are unrestored, the stress concentration caused by the cervical lesion may cause further deterioration of the tooth [9]. For this reason, in the presence of occlusal or fissure caries, this cavity design can be modified and extended to include a traditional endodontic access cavity which can be referred to as a bucco-occlusal access cavity (BOEC).

Finite element analysis (FEA) was first introduced originally in the field of engineering. It gained popularity in dentistry, especially modeling teeth, bone, tooth restorations, and nanocoatings on implants and devices [10, 11] due to its reproducible and numerical methodology [12]. In FEA, a physical model is divided into smaller elements called finite elements and then a mesh model of the structure is formed [11]. This method allows the generation of a virtual picture of the mechanical properties of the tooth and restoration [5] and it has been reported that the results of FEA studies affirm the results of laboratory-based studies [13]. It can overcome the limitation of the standardization of teeth because of possible variations in dentin mechanical properties, age, tooth extraction forces, storage time, and storage medium after extraction [14].

Mandibular premolars are inherently more susceptible to fracture due to the lingual orientation of these teeth, resulting in concentration of tensile stresses in the cervical section. This preference for structural compromise under stress makes them particularly important for study giving critical information about failure trends following different access cavity designs and restorations [15]. The load capacity and mechanical properties of mandibular premolars have been investigated in previous FEA and push-out studies [15–18]. However, knowledge on the effect of endodontic access cavity design on the load capacity of mandibular premolars under various occlusal forces is limited. Therefore, this study aimed to investigate the influence of conventional and modified endodontic access cavity designs on the mechanical properties of FEA single-rooted mandibular premolar models under simulated vertical and oblique occlusal forces. The null hypothesis is that the access cavity design has no effect on the stress distribution of a mandibular premolar tooth under static occlusal loads.

## Materials and methods

The study protocol was approved by university ethics committee (Approval no: 2019/642).

The manuscript of this laboratory study has been written according to Preferred Reporting Items for Laboratory studies in Endodontology (PRILE) 2021 guidelines [19].

An intact, mature, carious free, extracted single-rooted mandibular first premolar without any resorption was scanned with cone beam computed tomography (ILUMA, Orthocad, CBCT, 3 M Imtec, Oklahoma, USA) using the parameters of 90 kV, 12 mA, 75 μm slice thickness. A three-dimensional model with enamel, dentin and the cementum in a single structure was obtained by using Rhinoceros 4.0 Software (3670 Woodland Park Ave N, Seattle, WA 98,103 USA).

### Access cavity design

**Fig. 1:**
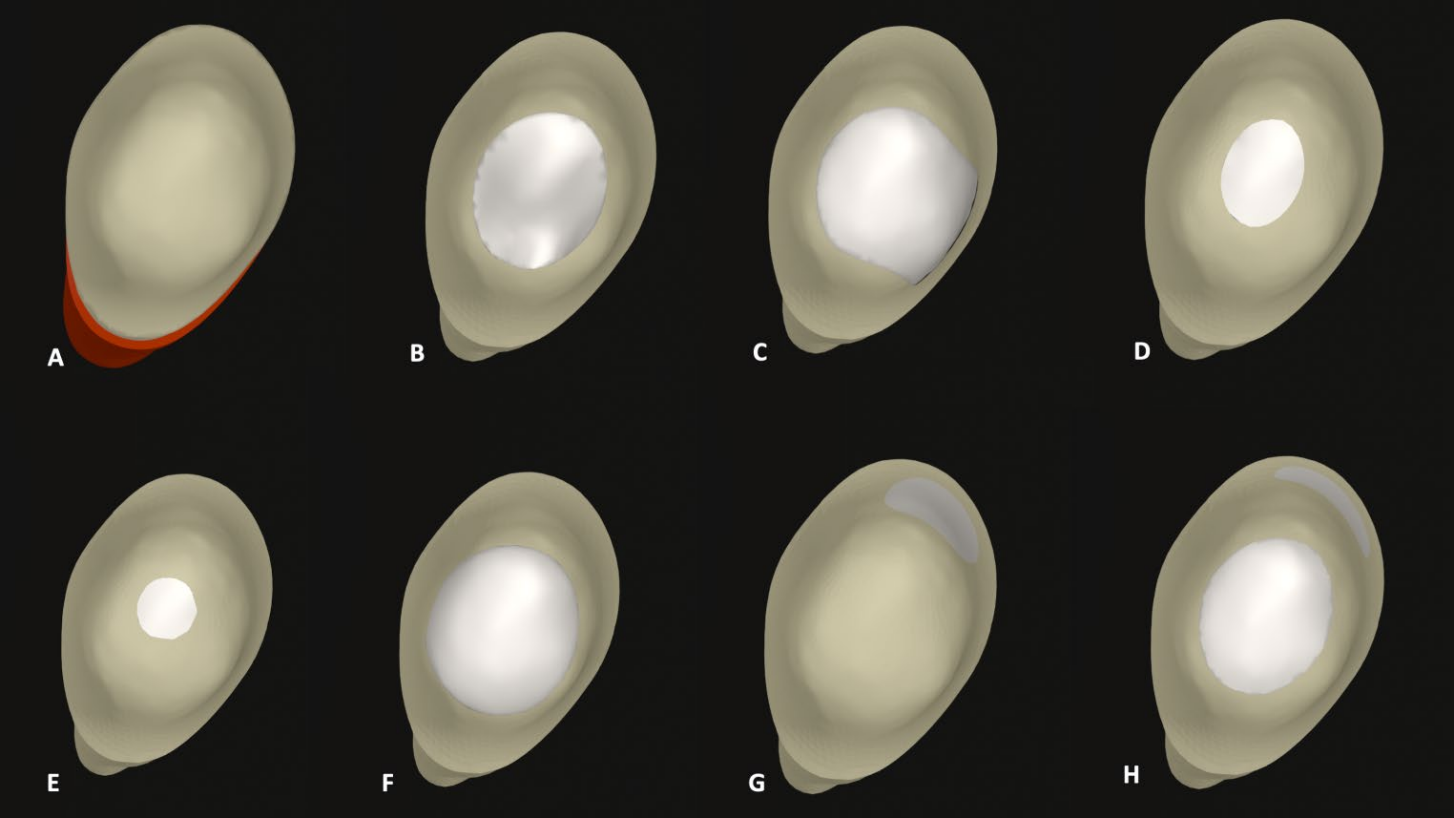
Digital experimental cavity designs: Control (**A**), TEC-I (**B**), TEC-II (**C**), CEC (**D**), NEC (**E**), Cd-EC (**F**), BEC (**G**) and BOEC (**H**), respectively

**Fig. 2:**
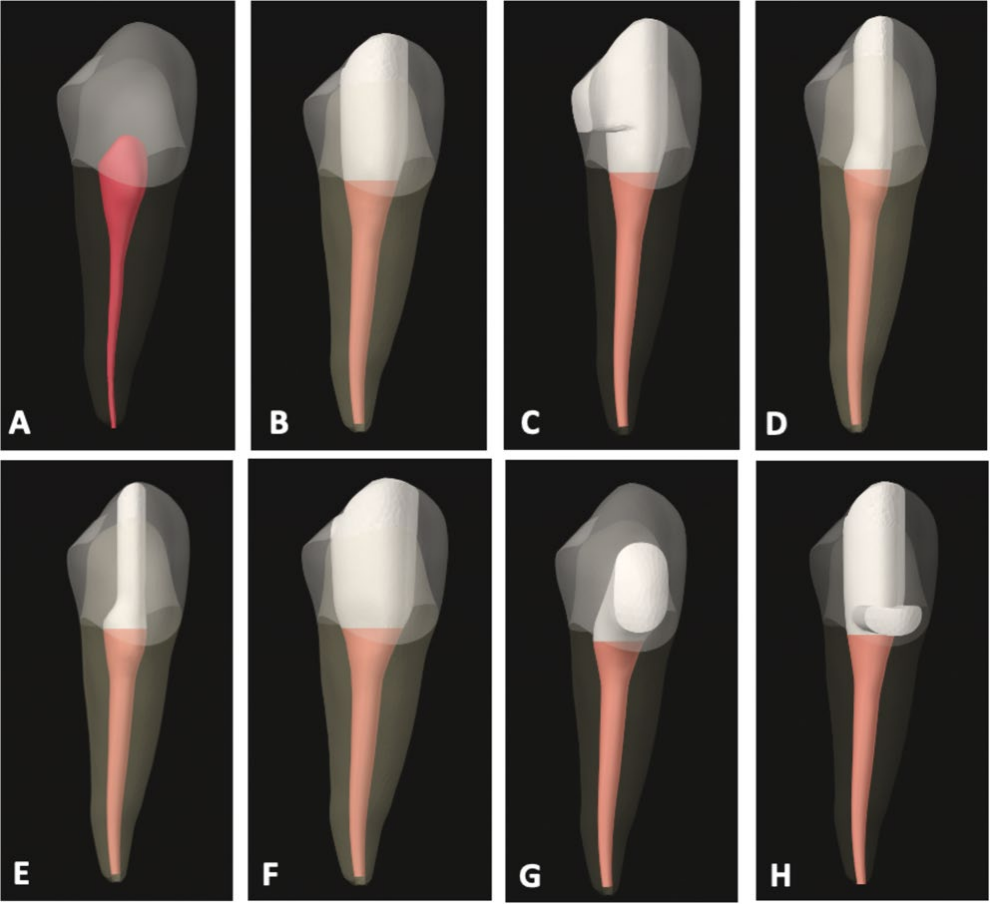
Lateral view of the models. Control model (**A**); TEC-I (**B**); TEC-II (**C**); CEC (**D**); NEC (**E**); Cd-EC (**F**); BEC (**G**) and BOEC (**H**), respectively

**Fig. 3:**
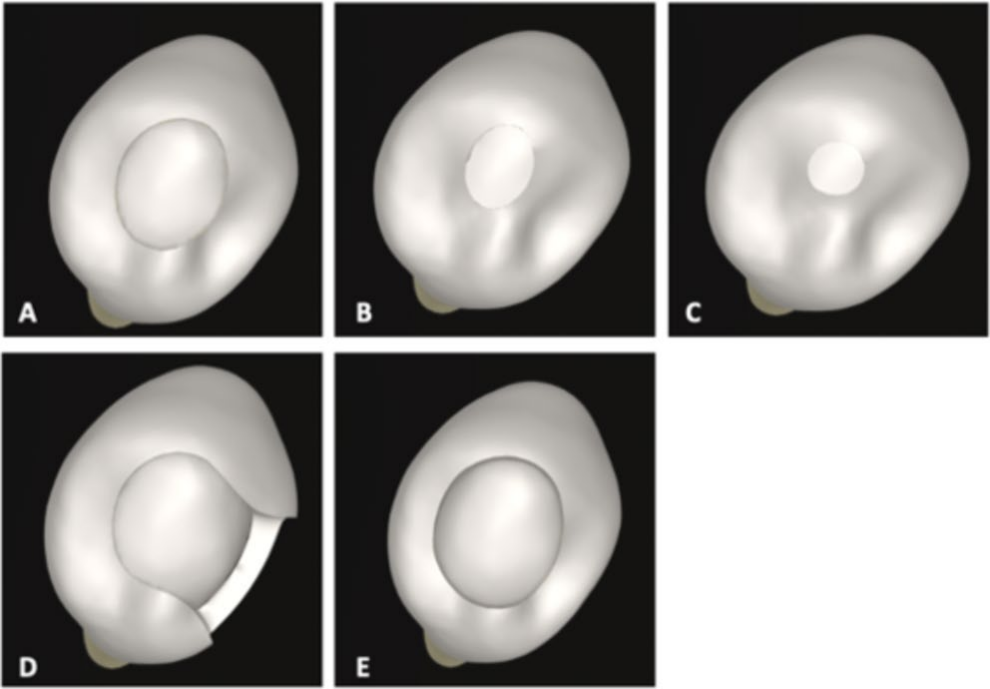
Occlusal view of the models. TEC-I (**A**); CEC (**B**); NEC (**C**); TEC-II (**D**); Cd-EC (**E**)

An intact tooth (Figs. 1A and 2A) and 7 different access cavity designs were modeled based on traditional, minimally invasive and modified principals. Occlusal view of the models were shown in Fig. 3.

To transfer the traditional cavity principals to the models, the traditional endodontic cavity (TEC-I) in which straight line access to the pulp chamber with complete deroofing was designed (Figs. 1B and 2B). The access cavity design in which the mesial marginal ridge was removed and thus converted to a Class-II restoration was named as TEC-II (Figs. 1C and 2C).

To simulate minimally invasive endodontic access cavities, a conservative access cavity (CEC) (Figs. 1D and 2D), a ninja access cavity (NEC) (Figs. 1E and 2E) and a buccal access cavity (BEC) (Figs. 1G and 2G) models were designed. In the CEC design, the cavity boundary started at the center of the occlusal surface down to the root canal orifice thus retaining part of the chamber roof [20] and lingual shelf [21]. The NEC model was designed with the same principles as the CEC, but the cavity was smaller yet still allowed observation of the root canal at different angles and preservation of the pulp chamber [20]. The BEC design was simulated with a diamater of 2 mm, centrally located in the mesio-distal direction in the buccal lower half of the crown. The access cavity was modified to provide access to the root canals from the buccal aspect.

In order to mimic clinical conditions, the modified endodontic access cavities were also supplemented with a caries-driven access cavity (Cd-EC) and a bucco-occlusal access cavity (BOEC). In the Cd-EC (Fig. 1E) design, the TEC-I design was enlarged on the occlusal surface allowing 2 mm of dentin to remain on the proximal margins [22]. The BOEC (Fig. 1H) model was the combination of BEC and TEC-I designs in which the occlusal access cavity was joined up with the BEC design.

### Root canal preparation

Root canal dimensions were simulated as size 40, 0.04 taper at 0.5 mm coronal to the apical foramen. The root canal was filled with simulated gutta-percha up to 2 mm from the root canal orifice [23, 24]. An endodontic sealer was not simulated in the FEA modelling [25]. The root canal orifice was filled with simulated flowable composite and then the entire access cavity was restored with composite resin. The volume of used composite in TEC-I, TEC-II, CEC, NEC, Cd-EC, BEC and BOEC designs was 60.146 mm^3^, 78.905 mm^3^, 26.724 mm^3^, 16.679 mm^3^, 76.103 mm^3^, 41.764 mm^3^ and 66.941 mm^3^, respectively.

### Set material properties

The thickness of the periodontal ligament, lamina dura and cortical bone was set to 0.2 mm, 0.3 mm and 2 mm, respectively [20, 26]. Cementum was modelled as 0.175 mm thick in the apical third and 0.038 mm thick in the coronal third. Cortical bone was designed as a 15 mm cube around the root starting 1.5 mm below the cementoenamel junction.

All models were designed in a three-dimensional format in the VRMesh Software and then imported into the Algor Fempro Software program for meshing. In accordance with previous studies, the teeth and materials were assumed to be homogeneous, linear, elastic, and isotropic [13]. The number of elements and nodes is summarized in Table 1. The elastic modulus and the Poisson ratio of the structures used in the FEA models were determined according to data derived from the literature [23, 27–31] and listed in Table 2.

**Table 1:** The number of nodes and elements of models

	Number		Control	TEC-I	TEC-II	CEC	NEC	Cd-EC	BEC	BOEC
Static I		Node	53,088	44,980	47,132	48,662	51,980	48,108	54,987	50,014
		Elements	252,167	220,218	227,589	237,918	249,666	231,205	265,277	236,785
Static II		Node	53,088	44,980	47,132	48,662	51,980	48,108	54,987	50,014
		Elements	252,167	220,218	227,589	237,920	249,665	231,205	265,277	236,785
Static III		Node	36,980	44,980	47,132	48,662	51,980	48,108	54,987	50,014
		Elements	198,253	220,213	227,589	237,920	249,666	231,217	265,277	236,772

**Table 2:** The mechanical characteristic of investigated material

Materials	Elastic modulus (E; MPa)	Poisson ration (µ)
**Enamel** (Sathorn et al., 2005)	84,100	0.33
**Dentin** (Sathorn et al., 2005)	18,600	0.31
**Periodontal Ligament** (Sathorn et al., 2005)	68.9	0.45
**Gutta-percha** (Helal & Wang, 2019)	140	0.40
**Cortical bone** (Huempfner-Hierl et al., 2014)	13,700	0.3
**Cancellous bone** (Huempfner-Hierl et al., 2014)	1370	0.3
**Composite resin** (Jiang et al., 2018)	12,000	0.3
**Flowable composite resin** (Jiang et al., 2018)	5100	0.27
**Cement** (Eskitaşçıoğlu et al., 2002)	6800	0.31
**Pulp** (Gale & Darvell, 1999)	3	0.45

### FEA

All the models were subjected to simulated static loads. Three different load types were applied to the models.

**Fig. 4:**
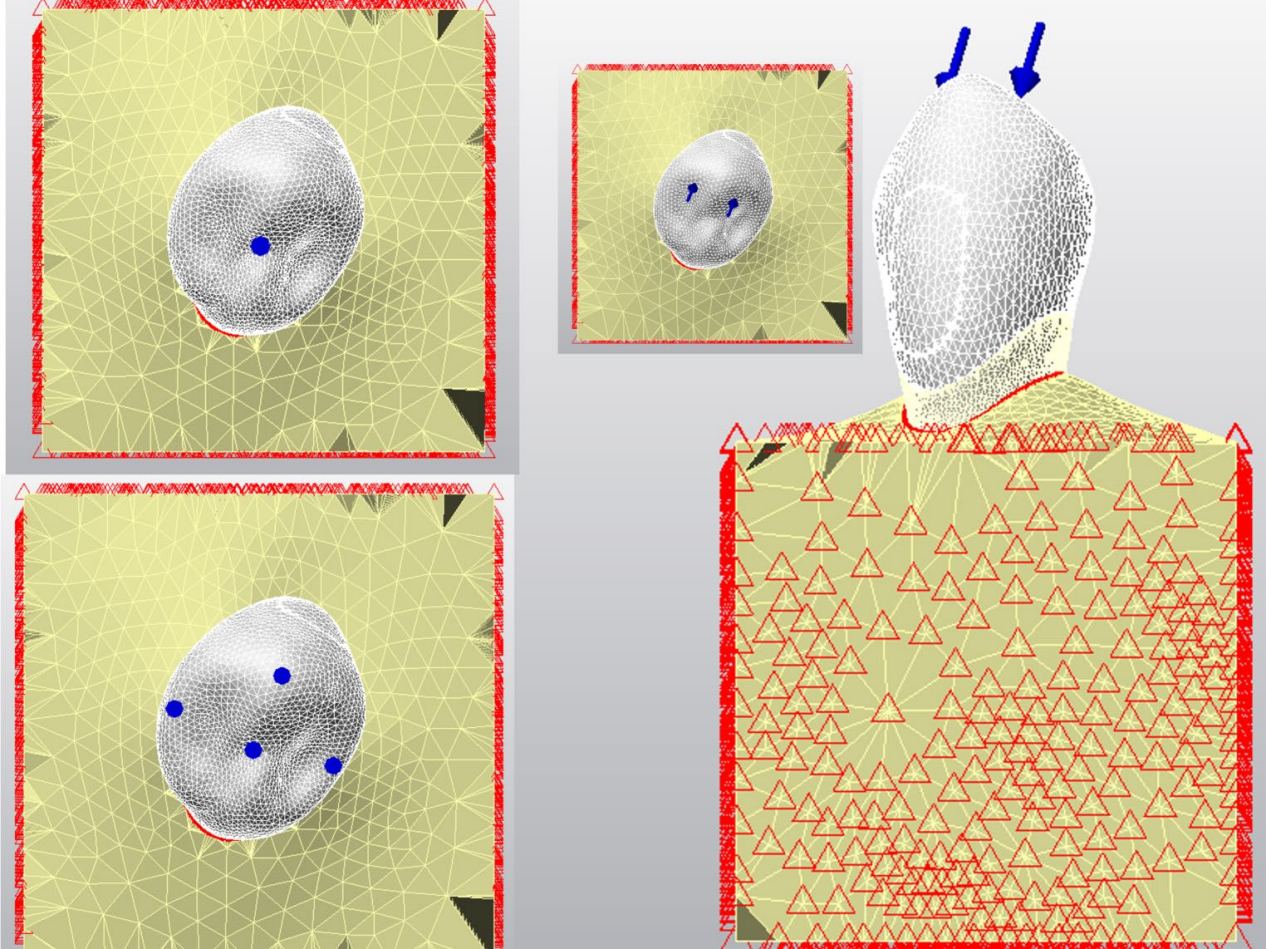
The load locations for single-point occlusal load; Static-I (**A**), multi-point occlusal load; Static-II (**B**) and multi-point oblique load; Static-III (**C**) forces from occlusal view. The lateral view of the Static III load is also presented in (**D**)

- For the Static-I load, a vertical load of 250 N was applied only from the central fossa. Because the load was applied form only one point, this type of load was defined as a “single-point vertical load” (Fig. 4A) [32].
- For Static-II load, a vertical occlusal load of 200 N (a total of 800 N) was applied from each of 4 points, namely the buccal cusp, central fossa and 2 marginal ridges. This type of load was defined as a “multi-point vertical load” (Fig. 4B) [33].
- For Static-III load, a total chewing load of 225 N was applied from two points (lingual surface of the buccal cusp) at an angle of 45° to the long axis of the tooth to simulate the intercuspation contact. This type of load was defined as a “multi-point oblique load” (Fig. 4C-D) [34].

For “multi-point load” types, the occlusal force was loaded not only from one point like in Static-I load but also from two or more points on occlusal surface like in Static- II and III loads. The load types are also shown in Fig. 4 with details.

Maximum von Mises (VM) Stress values and the stress distribution of the models under static loads were evaluated and compared.

## Results

The peak VM stress distribution on enamel and dentin is shown in Table 3. The maximum stress values were obtained on both enamel and dentin under Static-II load. Under all load types, the minimum stress distribution was observed in the control group (intact tooth), which was followed by CEC, NEC and BEC designs.

**Table 3:** The maximum Von mises (VM) stress values recorded in enamel and dentin of experimental models

		Control	TEC-I	TEC-II	CEC	NEC	Cd-EC	BEC	BOEC
Static I	Enamel	2576.78	1980.6	1587.57	1785.68	1685.78	1305.94	1791.19	1423.69
	Dentin	63.5812	212.135	51.3136	115.247	95.1722	95.724	60.2596	59.5486
Static II	Enamel	2787.45	2488.29	3211.45	7070.33	2396.69	3149.83	2106.84	2582.94
	Dentin	78.3298	191.574	162.725	115.58	139.783	114.573	95.1778	72.8548
Static III	Enamel	582.914	1251.03	1435.52	613.064	779.178	1110.47	616.137	1021.23
	Dentin	78.1796	88.1234	75.6949	98.8375	78.6388	98.1737	89.3717	141.512

**Fig. 5:**
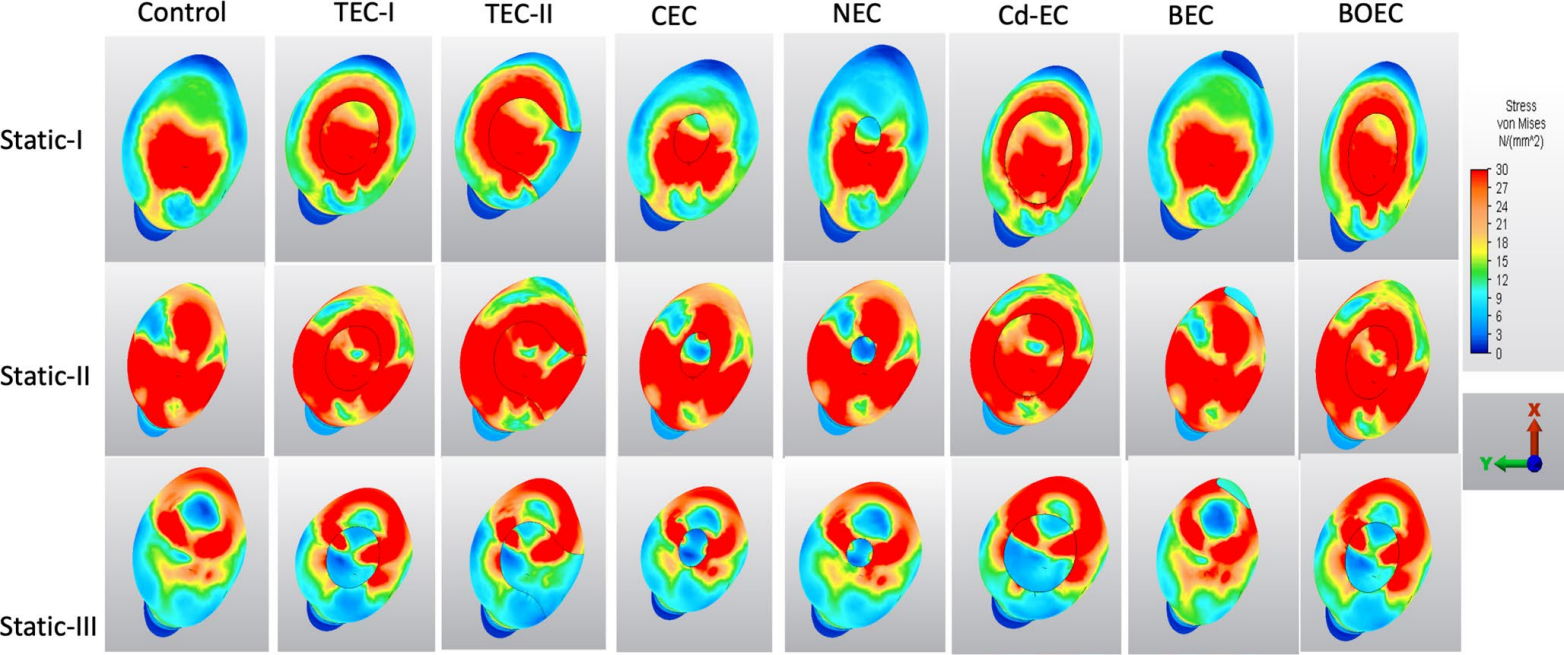
The stress distribution on the occlusal surface of the FEA models under Static-I, II and III loads

On the enamel surface, the stress mainly aggregated around the cavity margins, approximal surfaces and buccal side of the crown under Static-I, II and III loads, respectively (Fig. 5). The minimum stress distribution was observed in the control group and the maximum was in Cd-EC design followed by TEC-I and TEC-II under all load types. The minimum stress distribution among the experimental cavity designs was observed in CEC and NEC designs under Static II and III loads, and in BEC designs under Static I load. For the modified buccal access cavity groups, the stress distribution pattern under Static-II load was different. In the BEC design, the stress accumulated at the margins of the buccal cavity, while in the BOEC design, the stress was mainly concentrated around the lingual cusp.

**Fig. 6:**
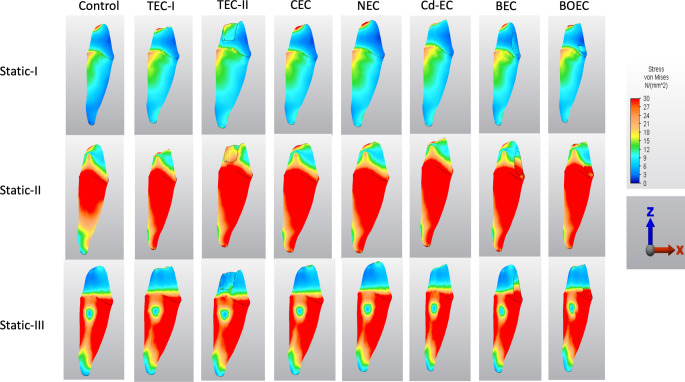
The stress distribution on the root, pericervical dentin and apex of the models

On the dentin surface, the stress distribution pattern on the root surface was similar for all cavity groups (Fig. 6). NEC and CEC designs were associated with less VM stress than the other experimental groups. The highest stress concentration on the root surface was detected on the TEC-II design and followed by the Cd-EC design under Static-I, II and III loads. Under single-point vertical loading, the stress was mostly concentrated in the lingual PCD area, while under multi-point vertical loading, the entire root surface was stress-loaded except for the lingual apical third of the root. For multi-point oblique forces, the stress was concentrated on the buccal and lingual surfaces of the root while decreasing in the middle third of the approximal surfaces. The BEC design had less stress concentration and distribution than the BOEC design under all load types.

## Discussion

The prognosis of root filled teeth depends on several factors, such as detection of the root canal orifices, the quality of chemo-mechanical canal preparation and filling of the root canals. The procedure causes a certain amount of tooth tissue loss which mainly occurs with the access cavity. In this study, the stress distribution of a single-rooted mandibular first premolar tooth with various endodontic access cavity designs was investigated under a range of simulated static occlusal loads. The novelty of the study is to test the mechanical performance of different access cavities, especially Cd-EC and BEC which were not evaluated before, by using an objective test, FEA. According to the results, minimal invasive cavity designs represented by NEC, CEC and BEC models were associated with less stress distribution on the tooth surface. In other words, the different access cavity designs showed different stress distributions throughout the dentin and enamel. In the light of these findings, the null hypothesis was rejected.

In the present study, the maximum VM stress was seen on enamel, rather than dentin under all types of loads. The main reason for this may be the mechanical and physical properties of these specific tissues. Both enamel and dentin play a crucial role in tooth fractures. However, the transmission of the load through these two tissues is different [35]. While enamel is the first tissue that encounters grinding stress with low tensile strength and high modulus of elasticity, the dentin absorbs the biting force that comes from the enamel [36]. This situation may explain the present results which were also supported by a previous study [37].

In this study, the type of endodontic access cavity appears to affect the stress distribution on the tooth surface. Among the experimental groups, NEC and CEC had the lowest stress distribution under all types of loads, except the Static-I load in which the BEC design had lower VM values. Many reports have concluded that the amount of tissue lost was directly related to the tooth strength against the mastication forces, which is also supported by the present findings [23, 24, 38]. Therefore, it is quite logical to observe less stress distribution associated with minimal invasive access cavities. On the other hand, the highest stress distribution was mainly observed in the Cd-EC design, followed by TEC cavities on the enamel surface. Considering this result, it can be concluded that while marginal tissue thickness was an important parameter in stress distribution, the loss of one marginal ridge had no significant effect on the enamel strength [38].

The pattern of stress distribution was affected by the direction and position of the occlusal load. [38, 39]. Benazzi et al. performed a FEA study with intact mandibular premolar models under different occlusal loads [15]. They reported that the tensile stress was mainly concentrated on the buccal side of the crown and the root whereas the minimum stress was observed on the lingual side of the root under Static-III load. In the present study, similar results were obtained with one exception. The lingual surface of the root also was associated with high-stress patterns in all experimental access cavity designs. So, the root filled single-rooted mandibular first premolar appears to be at risk of fracture from the lingual surface of the root, regardless of the access cavity design.

Non-carious cervical lesions are common on the buccal aspect of the crown of mandibular premolars. One of the hypotheses to explain this situation is that the high tensile stress occurs on the buccal wall of the crown under non-axial mastication forces [15], which will cause abfraction at the cervical region of the tooth [40]. This situation may generate two different approaches for access cavity preparation. The clinician creates either a BEC or BOEC (combine TEC design with Class V restoration) to reach the pulp chamber. In this study, the stress distribution for these two cavity designs was different. The BEC design, which was designed as a minimal invasive endodontic procedure, had less stress concentration and distribution than the BOEC design under all load types. This may indicate that the BEC is a good choice for a tooth with this kind of cervical lesion. However, the irrigating and shaping efficiency of the root canals, risk of file separation, centering ability, transportation, and procedural errors with BEC designs is unclear and must be investigated in future studies.

Teeth are exposed to a wide range of forces in the mouth. In order to predict the clinical behaviour and loading capacity of a tooth, static or dyanmic loading tests can be used. In the present study, while Static-I and II loads mimicked vertical mastication forces, Static-III represented an oblique mastication force [23]. When the direction of the load was changed from a single point and vertical to multipoint and oblique, the stress pattern on the root surface changed and increased substantially in all access cavity types. It can be interpreted that oblique and multipoint forces are more dangerous for mandibular premolar in terms of tooth fracture. This finding is also supported by previous studies [41, 42].

The limitation of the study must also be taken into consideration. Only one specific mandibular premolar model was evaluated using a constant and standard root canal size. It is unknown whether any difference in root canal size and taper may affect the stress distribution on the tooth surface. In addition, the stress distribution was analyzed only under static loads. However, it was reported in a previous study that the fracture strength of cementum was different under static and fatigue load [42, 43]. Therefore, the load capacity of the tooth and consequently the probabilty of survival may vary under a range of forces. Moreover, usage of fiber-reinforced composite filling, post-core restoration, adhesion quality of restoration, tensile stress arise from polymerization shrinkage, cusp coverage and/or cusp reduction in root filled mandibular premolars may also have an impact on load capacity [44–46].

Finite Element Analysis (FEA) offers significant benefits. It enables researchers to obtain stress distributions within intricate structures under various conditions, which can be challenging to achieve through laboratory experiments (23). Clinical or experimental research often faces numerous confounding factors, such as operator or observer bias, differences in tooth anatomies, operational flaws, and equipment calibration issues, among others [23, 47]. Despite the invaluable insights provided by FEA in dental research, it is important to acknowledge the inherent limitations of this method. FEA, being a computerized virtual simulation, cannot fully replicate the complexities of the clinical environment. The assumption of homogeneous, isotropic, and linear mechanical properties in the materials used in FEA models oversimplifies the reality, as dental structures such as the tubular structure of dentin and the dentin-enamel junction are functionally graded materials exhibiting varying elastic moduli and creep-related behaviors. This discrepancy underscores the need for cautious interpretation of FEA results and highlights the importance of complementing virtual models with empirical clinical data [47, 48]. Besides, it should be kept in mind that various controlled and uncontrolled factors can influence static load experiments, such as the loading position, the angle of the load, whether a full-coverage restoration is present, the age of the dentin, the extent of hard tissue loss, and collagen degradation, among other factors [49].

## Conclusion

Within the limitation of the study, these are the main conclusions:

1. Minimally invasive cavities relieved the stress distribution.
2. CEC, NEC and BEC designs were associated with lower stress distribution than other experimental cavity designs on the root and crown of a root filled single-rooted first premolar tooth. Therefore, it can be concluded that the BEC design could be a good alternative in the presence of cervical lesions.
3. The application of oblique and multipoint forces were identified as critical loads that impact the failure probabilities of a root filled single-rooted first premolar tooth.

Further clinical and laboratory studies are needed to evaluate the effect of restoration and dynamic loads on the mechanical behavior of mandibula premolars.

## Data Availability

No datasets were generated or analysed during the current study.

## References

[1] Clark D, Khademi J (2020) Modern molar endodontic access and directed dentin conservation. Dent Clin North Am 54:249–273 10.1016/j.cden.2010.01.001 20433977

[2] Aazzouzi-Raiss K, Raminez-Munoz A, Mendez P et al (2023) Effects of conservative access and apical enlargement on shaping and dentin preservation with traditional and modern instruments: a micro-computed tomographic study. J Endod 49:430–437 36646164 10.1016/j.joen.2023.01.004

[3] Boveda C, Kishen A (2015) Contracted endodontic cavities: the foundation for less invasive alternatives in the management of apical periodontitis. Endod Top 33:169–186 10.1111/etp.12088

[4] Buchanan GD, Gamieldien MY, Fabris-Rotelli I et al (2022) A study of mandibular premolar root and canal morphology in a black South African population using cone-beam computed tomography and two classification systems. J Oral Sci 64:300–306 36089376 10.2334/josnusd.22-0239

[5] Shubhashini N, Meena N, Shetty A et al (2008) Finite element analysis of stress concentration in Class V restorations of four groups of restorative materials in mandibular premolar. J Conserv Dent 11(3):121–126 20142899 10.4103/0972-0707.45251 PMC2813101

[6] Cohen S, Burns RC (2002) Pathways of the pulp, 8th edn. CV Mosby, St.Louis, p 174

[7] Du Y, Lee Angeline HC, Zhang C (2013) Mandibular first premolar with four canals. JICD 4:64–66 23382063 10.1111/j.2041-1626.2012.00127.x

[8] Wilcox LR, Walton RE (1987) The shape and location of mandibular premolar access openings. Int Endod J 20:223–227 3481784 10.1111/j.1365-2591.1987.tb00618.x

[9] Pai S, Bhat V, Patil V et al (2020) Numerical three-dimensional finite element modeling of cavity shape and optimal material selection by analysis of stress distribution on class V cavities of mandibular premolars. J Int Soc Prev Community Dent 10(3):279–285 32802773 10.4103/jispcd.JISPCD_75_20 PMC7402252

[10] Choi AH, Conway RC, Bennıssan B (2014) Finite-element modeling and analysis in nanomedicine and dentistry. Nanomedicine 9(11):1681–1695 25321169 10.2217/nnm.14.75

[11] Ince Yusufoğlu S, Sarıçam E, Özdoğan MS (2023) Finite Element Analysis of Stress Distribution in Root canals when using a Variety of Post systems Instrumented with different Rotary systems. Ann Biomed Eng ;1–13 10.1007/s10439-023-03145-w 36705864

[12] Chien PHY, Walsh LJ, Peters OA (2021) Finite element analysis of rotary nickel–titanium endodontic instruments: a critical review of the methodology. Eur J Oral Sci 129:e12802 34105190 10.1111/eos.12802

[13] Ausiello P, Apicella A, Davidson CL (2002) Effect of adhesive layer properties on stress distribution in composite restorations–a 3D finite element analysis. Dent Mater 18:295–303 11992906 10.1016/S0109-5641(01)00042-2

[14] Versiani MA, Cavalcante DM, Belladonna FG, Silva EJNL, Souza EM, De-Deus G (2022) A critical analysis of research methods and experimental models to study dentinal microcracks. Int Endod J 55:178–226 34743355 10.1111/iej.13660

[15] Pai S, Naik N, Patil V et al (2019) Evaluation and comparison of stress distribution in restored cervical lesions of mandibular premolars: three-dimensional finite element analysis. J Int Soc Prev Community Dent 9:605–611 32039081 10.4103/jispcd.JISPCD_301_19 PMC6905322

[16] Benazzi S, Grosse IR, Gruppioni G et al (2014) Comparison of occlusal loading conditions in a lower second premolar using three-dimensional finite element analysis. Clin Oral Invest 18:369–375 10.1007/s00784-013-0973-8 23504207

[17] Chlup Z, Zizka R, Kania J et al (2017) Fracture behavior of teeth with conventional and mini-invasive access cavity designs. J Eur Ceram Soc 37:4423–4429 10.1016/j.jeurceramsoc.2017.03.025

[18] Wang Z, Fu B (2022) Minimum residual root dentin thickness of mandibular premolars restored with a post: a finite element analysis study. J Prosthet Dent. 10.1016/j.prosdent.2022.03.024. (in press) 35489836 10.1016/j.prosdent.2022.03.024

[19] Nagendrababu V, Murray PE, Ordinola-Zapata R et al (2021) PRILE 2021 guidelines for reporting laboratory studies in Endodontology: A consensus‐based development. Int Endod J 54:1482–1490 33938010 10.1111/iej.13542

[20] Liu Y, Liu H, Fan B (2021) Influence of cavity designs on fracture behavior of a mandibular first premolar with a severely curved h-shaped canal. J Endod 47:1000–1006 33775730 10.1016/j.joen.2021.03.012

[21] Plotino G, Grande NM, IsufiA et al (2017) Fracture strength of endodontically treated teeth with different access cavity designs. J Endod 43:995–1000 28416305 10.1016/j.joen.2017.01.022

[22] Chaniotis A, Plotino G (2021) Minimally invasive access to the root canal system. Minim Invasive Approaches Endodontic Pract, 45–65

[23] Jiang Q, Huang Y, Tu XR, Li Z, He Y, Yang X (2018) Biomechanical properties of first maxillary molars with different endodontic cavities: a finite element analysis. J Endod 448:1283–1288 10.1016/j.joen.2018.04.004 29910031

[24] Elkholy MM, Nawar NN, Ha WN et al (2021) Impact of Canal Taper and Access Cavity Design on the Lifespan of an endodontically treated Mandibualr Molar: a finite element analysis. J Endod 47:1472–1480 34139264 10.1016/j.joen.2021.06.009

[25] Zelic K, Vukicevic A, Jovicic G et al (2015) Mechanical weakening of devitalized teeth: three-dimensional finite element analysis and prediction of tooth fracture. Int Endod J 48:850–863 25243348 10.1111/iej.12381

[26] Ichim I, Schmidlin PR, Kieser JA et al (2007) Mechanical evaluation of cervical glassionomer restorations: 3D finite element study. J Dent 35:28–35 16782259 10.1016/j.jdent.2006.04.003

[27] Eskitascioglu G, Belli S, Kalkan M (2002) Evaluation of two post core systems using two different methods (fracture strength test and a finite elemental stress analysis). J Endod 28:629–633 12236304 10.1097/00004770-200209000-00001

[28] Gale MS, Darvell BW (1999) Thermal cycling procedures for laboratory testing of dental restorations. J Dent 27:89–99 10071465 10.1016/S0300-5712(98)00037-2

[29] Sathorn C, Palamara JE, Palamara D, Messer HH (2005) Effect of root canal size and external root surface morphology on fracture susceptibility and pattern: a finite element analysis. J Endod 3:288–289 10.1097/01.don.0000140579.17573.f7 15793386

[30] Huempfner-Hierl H, Schaller A, Hemprich A, Hier T (2014) Biomechanical investigation of naso-orbitoethmoid traumaby finite element analysis. Brit J Oral Maxillofac Surg 52:850–853 25138612 10.1016/j.bjoms.2014.07.255

[31] Helal MA, Wang Z (2019) Biomechanical assessment of restored mandibular molar by endocrown in comparison to a glass fiber post-retained conventional crown: 3D finite element analysis. J Prosthodont 28(9):988–996 29067737 10.1111/jopr.12690

[32] Fernandes CP, Glantz PO, Svenssom SA, Bergmark A (2003) A novel sensor for bite force determinations. Dent Mater 19:118–126 12543117 10.1016/S0109-5641(02)00020-9

[33] Zhang Y, Liu Y, She Y, Liang Y, Xu F, Fang C (2019) The effect of endodontic access cavities on fracture resistance of first maxillary molar using the extended finite element method. J Endod 45(3):316–321 30803539 10.1016/j.joen.2018.12.006

[34] Liu B, Lu C (2011) The effects of adhesive type and thickness on stress distribution in molars restored with all-ceramic crowns. J Prosthodont 20:35–44 21073593 10.1111/j.1532-849X.2010.00650.x

[35] Benazzi S, Kullmer O, Grosse I et al (2012) Brief communication: comparing loading scenarios in lower first molar supporting bone structure using 3D finite element analysis. Am J Phys Anthropol 147:128–134 21952986 10.1002/ajpa.21607

[36] Chun KJ, Lee JY (2014) Comparative study of mechanical properties of dental restorative materials and dental hard tissues in compressive loads. J Dent Biomech 5:1758736014555246 25352921 10.1177/1758736014555246 PMC4209892

[37] Gönder HY, Demirel MG, Mohammadi R, Alkurt S, Fidancioğlu YD, Yüksel IB The effects of using cements of different thicknesses and Amalgam restorations with different Young’s Modulus values on stress on DentalTissue: An Investigation using finite element analysis. Coatings 2023;13,6.

[38] Ballester B, Giraud T, Aly Ahmed HM et al (2021) Current strategies for conservative endodontic access cavity preparation techniques—systematic review, meta-analysis, and decision-making protocol. Clin Oral Invest 25:6027–6044 10.1007/s00784-021-04080-7 34623506

[39] Benazzi S, Kullmer O, Grosse IR et al (2011) Using occlusal wear information and finite element analysis to investigate stress distributions in human molars. J Anat 219:259–272 21615398 10.1111/j.1469-7580.2011.01396.x PMC3171771

[40] Grippo JO (1991) Abfractions: a new classification of hard tissue lesions of teeth. J Esthet Dent 3:14–19 1873064 10.1111/j.1708-8240.1991.tb00799.x

[41] Palamara D, Palamara JEA, Tyas MJ et al (2000) Strain patterns in cervical enamel of teeth subjected to occlusal loading. Dent Mater 16:412–419 10967190 10.1016/S0109-5641(00)00036-1

[42] Zhu J, Rong Q, Wang X et al (2017) Influence of remaining tooth structure and restorative material type on stress distribution in endodontically treated maxillary premolars: a finite element analysis. J Prosthet Dent 1175:646–655 10.1016/j.prosdent.2016.08.023 27881319

[43] Ren LM, Wang WX, Takao Y, Chen ZX (2010) Effects of cementum–dentine junction and cementum on the mechanical response of tooth supporting structure. J Dent 38:882–891 20696202 10.1016/j.jdent.2010.07.013

[44] Elayouti A, Serry MI, Gerstorfer G et al (2011) Influence of cusp coverage on the fracture resistance of premolars with endodontic access cavities. Int Endod J 44:543–549 21276020 10.1111/j.1365-2591.2011.01859.x

[45] Ausiello P (2020) Stress distributions for Hybrid Composite Endodontic Post designs with and without a Ferrule: FEA Study. Polym (Basel) 16(8):1836 10.3390/polym12081836 PMC7465202 32824363

[46] da Rocha DM (2019) Effect of the restorative technique on load-bearing capacity, cusp deflection, and stress distribution of endodontically-treated premolars with MOD restoration. Restor Dent Endod 7(443):e33 10.5395/rde.2019.44.e33 PMC6713078 31485429

[47] Trivedi S (2014) Finite element analysis: a boon to dentistry. J Oral Biology Craniofac Res 4:200–203 10.1016/j.jobcr.2014.11.008 PMC4306993 25737944

[48] Abdelfattah RA, Nawar NN, Kataia EM, Saber SM (2024) How loss of tooth structure impacts the biomechanical behavior of a single-rooted maxillary premolar: FEA. Odontology 112(1):279–286 37394683 10.1007/s10266-023-00829-6 PMC10776703

[49] Mireku AS, Romberg E, Fouad AF, Arola D (2010) Vertical fracture of root filled teeth restored with posts: the effect of patient age and dentine thickness. Int Endod J 43:218–225 20158533 10.1111/j.1365-2591.2009.01661.x PMC3353984

